# A Chromate-Free and Convenient Route to Fabricate Thin and Compact Conversion Coating for Corrosion Protection on LZ91 Magnesium Alloy

**DOI:** 10.3390/nano12091614

**Published:** 2022-05-09

**Authors:** Chun-Wei Chen, Salim Levent Aktug, Chin-Jou Chang, Yueh-Lien Lee, Ming-Der Ger, Shun-Yi Jian

**Affiliations:** 1 Department of Chemical & Materials Engineering, Chung Cheng Institute of Technology, National Defense University, Taoyuan 33551, Taiwan; cwchen860510@gmail.com (C.-W.C.); asd870911@gmail.com (C.-J.C.); mingderger@gmail.com (M.-D.G.); 2 Department of Materials Science and Engineering, Gebze Technical University, Gebze 41400, Kocaeli, Turkey; salimleventaktug@gmail.com; 3 Department of Engineering Science and Ocean Engineering, National Taiwan University, Taipei 10617, Taiwan; yuehlien@ntu.edu.tw; 4 System Engineering and Technology Program, National Chiao Tung University, Hsin-Chu 300, Taiwan; 5 Department of Material Engineering, Ming Chi University of Technology, New Taipei City 24301, Taiwan

**Keywords:** LZ91 magnesium alloy, acid pickling pretreatment, conversion coating, microstructure, corrosion resistance

## Abstract

This study characterizes and determines the corrosion resistance of Mn-Ce conversion coated LZ91 magnesium alloy that undergoes pretreatments. It is challenging to process large and curved workpieces in the industry because the geometric shapes are complex if they are mechanically ground. This study uses acid pickling instead of mechanical grinding, and a nitric acid solution is used for pickling. After pretreatments, the samples are immersed for 30 s in a conversion coating solution containing 0.1 M KMnO_4_ and 0.025 M Ce(NO_3_)_3_ with a pH of 1.5, as demonstrated in previous studies by the authors. The microstructure of the coating layer and electrochemical behavior of conversion coated samples exposed to 3.5 wt.% NaCl solution are studied. The corrosion behavior of Mn-Ce conversion coating specimens is determined using a salt spray test (SST). Scanning electron microscopy (SEM), energy dispersive X-ray spectrometry (EDS), and X-ray photoelectron spectroscopy (XPS) are used to analyze the interface between the coating layer and the underlying magnesium substrate and to investigate the microstructure of the specimens. The roughness of the coatings is measured using 3D white light interferometry. The results show that the deteriorated area ratio for conversion coated LZ91 decreases to less than 5% after 72 h of SST exposure, and the corrosion resistance is improved 2.25 times with the Mn-Ce conversion coating on LZ91 magnesium alloy.

## 1. Introduction

Magnesium (Mg) alloys are one of the lightest metallic structures and are widely used for lightweight industrial applications, and their density is only around 1.74 g/cm^3^. Magnesium alloys occupy an important position in many engineering applications where lightness is the distinguishing feature due to their good ductility, tensile strength, wear resistance, impact resistance, and biocompatibility, but its resistance to corrosion is poor, so surface treatment is required to improve corrosion ability and expand the application areas of the alloy [1,2,3,4,5].

Common surface modification treatments for magnesium alloys include electroplating [6,7,8], electroless plating [9,10,11,12], anodizing [13,14] and physical vapor deposition (PVD) [15], and conversion coating [16,17,18,19]. The characteristics of each coating vary as coatings are often specific to the industrial processes in which the material is used. Compared to electroplating, electroless plating, and PVD treatments, conversion coating is a cheaper, more efficient, and advantageous manufacturing process for magnesium alloys due to no necessity for particular instruments or unique specifications [16,17]. Another advantage of conversion coating is in paint applications, which gives better adhesion strength than anodized surfaces. This benefit provides good electrical conductivity and electromagnetic shielding for 3C (Computer, Communication, and Consumer Electronics) products and other related applications [18,19].

Conversion coating involves a chemical or electrochemical process for which magnesium metal is dissolved and reacted with ions in acidic or alkaline solutions, and insoluble compounds are deposited on the magnesium surface to protect the substrate [20,21,22]. The composition of coatings includes magnesium hydroxides and metal oxides. The oxide-coated surface acts as a barrier layer to prevent aggressive media from diffusing into the substrate and accordingly increases the corrosion resistance of the material. Hexavalent chromium conversion coatings are widely used in industry, but they are harmful to the environment and the human body, so a RoHS (restriction of hazardous substances) directive means that many countries have banned hexavalent chromium and other conversion coating treatments, such as phosphate [23,24,25], manganate [26,27], Stannate [28,29] and rare earth salts [30,31,32].

Previous studies [33,34] showed that a KMnO_4_ conversion coating with added cerium provides better corrosion resistance and has a more compact coating structure due to increased chemical reactivity. Additionally, this Mn-Ce conversion coating treatment apparently showed an excellent adhesion, and, most importantly, the dissolved Ce(IV) ions in the coating provide unique corrosion protection known as self-healing, which means the coating layer itself can regenerate a new one when damaged [35]. However, test specimens are ground in a laboratory, but industrial applications involve many larger and curved workpieces. Chemical processes will eventually replace with mechanical grinding [36,37,38,39,40]. This study determines the effect of an acid pickling pretreatment on the nucleation and microstructure of a Mn-Ce conversion coating on LZ91 Mg alloy. A salt spray test (SST) is used to determine the corrosion resistance of Mn-Ce conversion coated samples subjected to different acid pickling pretreatments.

## 2. Experimental Procedure

### 2.1. Materials and Preparation of Specimens

A commercial cold-rolled LZ91 Mg alloy plate was used for this study. The composition was 9.57 wt.% of Li, 1.04 wt.% of Zn, and 0.25 wt.% of Al and bal. Mg. The thickness of all substrates was 1.5 mm × 50 mm × 25 mm. Before conversion coating, these substrates were subjected only to acid pickling pretreatment for LZ91 Mg alloy. The specimens were ground using 400# and 600# grit SiC paper to achieve a uniform surface, ultrasonically degreased in acetone, and dried in air to remove the metal oxides/hydroxides. The specimens were placed into an alkaline cleaning solution with 50 g/L sodium hydroxide (NaOH) and 10 g/L trisodium orthophosphate (Na_3_PO_4_·12H_2_O) at 65 °C for 10 min. DI water was used to clean the specimens, and they were then dried in a stream of air [36,37,41]. After alkaline cleaning, 5% nitric acid was used for the acid pickling pretreatment at room temperature for 30 s, 60 s, 90 s, and 120 s.

### 2.2. Conversion Coating Treatment

The conversion solution is composed of 0.1 M potassium permanganate (KMnO_4_) and 0.25 M cerium nitrate (Ce(NO_3_)_3_) and was adjusted to a pH = 1.5 with H_2_SO_4_ [33,34,35]. Acid pickling pretreatment of the specimens removes the hydroxide/oxide layer [42,43,44]. When the specimens are immersed in a concentrated acid solution, the substrate surface dissolves rapidly and is exposed to the conversion coating bath. After conversion coating, the specimens were immediately rinsed thoroughly with deionized water and dried in a stream of air at room temperature.

### 2.3. Coating Characterization

Scanning electron microscopy (SEM) using a JEOL JSM-IT100 microscope equipped with energy-dispersive spectrometer (EDS) was used to determine the morphology of the surface of the conversion coatings. FIB-SII 3050SE (Focused ion beam) was used for sample cutting. A transmission electron microscope (TEM) Philips/FEI Tecnai F30 (Hillsboro, OR, USA) with an acceleration voltage from a LaB6 gun of 300 keV was used. The depth profile was measured using a 3 keV Ar^+^ ion beam rastered with an incidence angle of 45°, and the binding energy of the various species in the coating was measured using X-ray photoelectron spectroscopy (XPS, VGS Thermo K-Alpha, Thermo Fisher Scientific Inc., Waltham, MA, USA). The chamber pressure for depth profiling was about 10^−7^ Torr, and the sputtering rate was estimated to be 30 nm/min for SiO_2_. The 3D white light interferometry (Chroma 7503, Chroma Ate Inc., Taoyuan, Taiwan) was used to determine the surface roughness of the samples. Contact angle measurement was utilized by a contact angle goniometer (FACE CA-5 150, Tantec, Chicago, IL, USA) under room temperature to examine the surface wettability. The quantitative elemental composition of various sample types can be provided using inductively coupled plasma optical emission spectroscopy (ICP-OES, Optima 2000 DV, PerkinElmer, Waltham, MA, USA).

### 2.4. Corrosion Behavior of the Coating

The corrosion resistance of the LZ91 plates was determined using potentiodynamic polarization in a 3.5 wt.% sodium chloride (NaCl) solution at 25 °C using a Potentiostat (VersaSTAT4, Princeton Applied Research, Ametek Inc., Berwyn, PA, USA). A Hg/KCl electrode was used as a reference, and a platinum plate served as the auxiliary electrode. The exposed surface area of the specimen was1.77 cm^2^. Each specimen was immersed in the test solution for 20 min to achieve a steady open circuit potential (OCP) prior to the polarization scan. The potentiodynamic polarization measurement was conducted by sweeping the potential between −300 mV and +500 mV vs. the OCP at a scan rate of 1 mV s^−1^ after the steady OCP was achieved.

An SST was used to determine the corrosion resistance of specimens by placing them in a salt spray chamber with a tilting angle of 30° and exposing them to 5 wt.% NaCl fog for 72 h, according to the ASTM B117-03 standard [45]. The samples coated on LZ91 measured as 50 × 25 × 1.5 mm. The degree of oxidation was classified by the ASTM D610-08 standard to calculate the percentage of corrosion for the conversion coatings [46]. The value for each electrochemical test is expressed as the average ± the standard deviation for three measurements, and SSTs were performed in triplicate to confirm the reproducibility of the results.

## 3. Results and Discussion

### 3.1. The Properties of the Specimens

**Figure 1:**
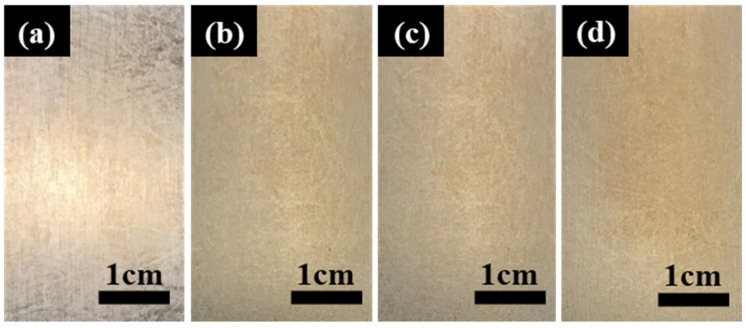
Optical images of LZ91 Mg alloy after acid pickling pretreatment for (**a**) 30 s, (**b**) 60 s, (**c**) 90 s, and (**d**) 120 s.

Figure 1 shows the acid pickling pretreatment using 5% nitric acid on the LZ91 Mg alloy surface. The surface is slightly yellowish-brown. As time increases, the surface of the sample becomes smoother. The grinding scratches on the sample are removed and cannot be observed visually.

**Figure 2:**
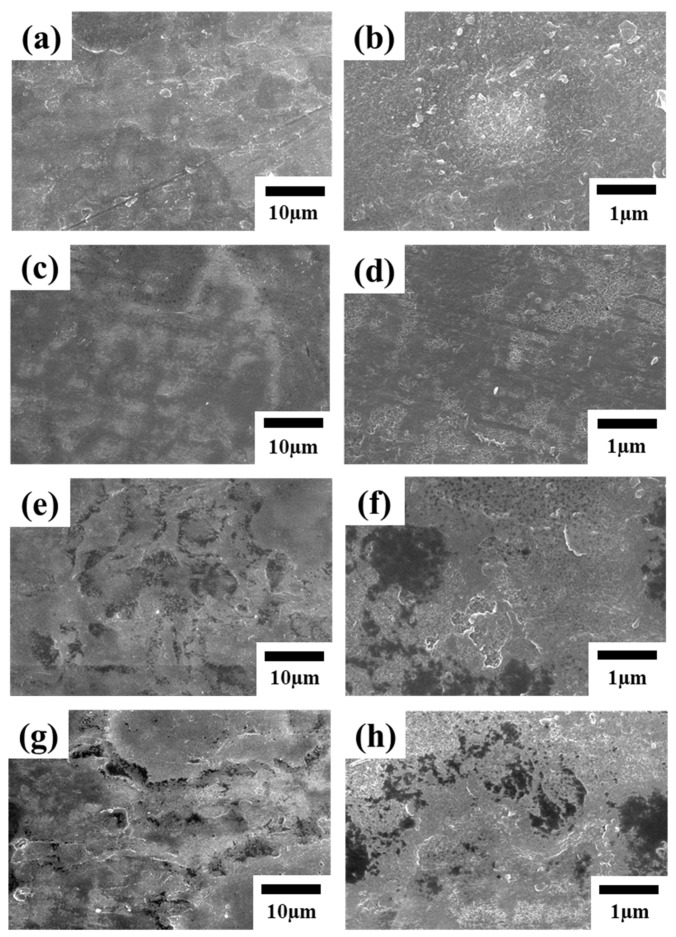
Surface morphology of LZ91 Mg alloy after acid pickling pretreatment for (**a**,**b**) 30 s, (**c**,**d**) 60 s, (**e**,**f**) 90 s, and (**g**,**h**) 120 s.

Figure 2 shows the SEM microstructure of LZ91 Mg alloy for different immersion times in the acid pickling pretreatment. After acid pickling pretreatment for 30 s, the surface is homogeneous and slightly undulated (Figure 2a,b). The surface becomes more homogeneous and has irregular light and dark patches, as shown in Figure 2e,g.

**Figure 3:**
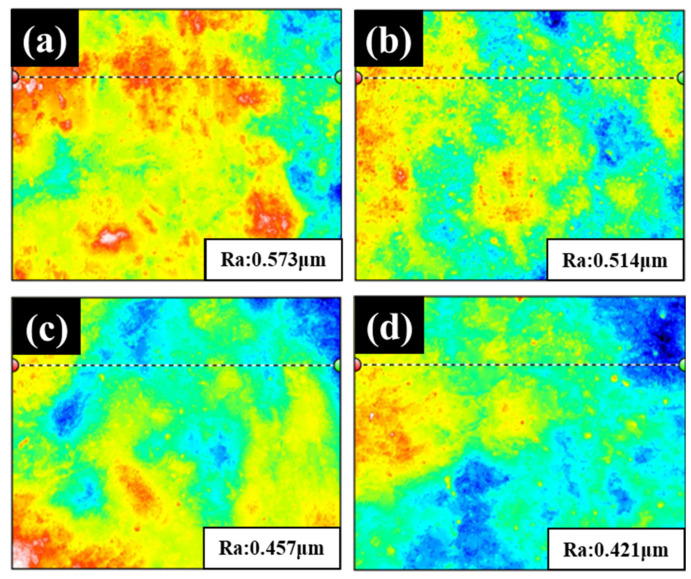
Three-dimensional white light diagram of LZ91 Mg alloy after acid pickling pretreatment for (**a**) 30 s, (**b**) 60 s, (**c**) 90 s, and (**d**) 120 s.

The surface roughness results for 3D white light interferometry are shown in Figure 3. The SEM images show that the surface is similar to a 3D white light diagram, with an average surface roughness between 0.573 μm and 0.421 μm. The roughness decreases when the specimen is immersed for more than 30 s because the β phase corrodes more quickly because the α phase corrodes more slowly than the β phase at different rates [19,34,47]. Figure 2c,d show obvious filament corrosion. As the immersion time increases to 90 s and 120 s, the surface becomes rougher because the β phase continues to corrode. There is a lower roughness after immersion for 90 s and 120 s because of the different corrosion rates, so there is less lithium β phase.

**Figure 4:**
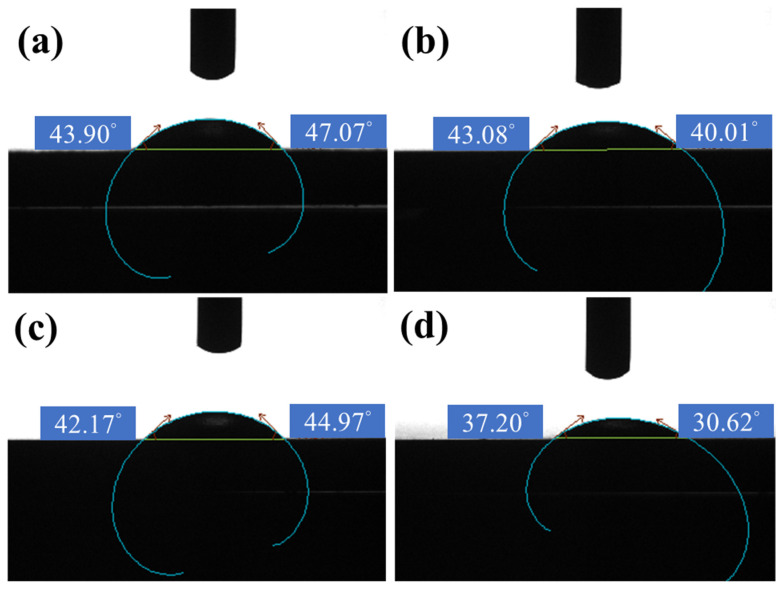
The contact angle for LZ91 Mg alloy after acid pickling pretreatment for (**a**) 30 s, (**b**) 60 s, (**c**) 90 s, and (**d**) 120 s.

Figure 4 shows the contact angle for LZ91 Mg alloy after acid pickling pretreatment at different times. SEM images of the microstructure and surface roughness show that the contact angle decreases as time increases. The contact angle measured at the 30 s immersion time is lower than that measured at 60 s and 90 s. However, after acid pickling pretreatment for 120 s, the contact angle is slightly less because there is a larger area of filiform corrosion at 120 s (Figure 2g); thus, the surface exhibits a bit more hydrophilic interaction.

**Figure 5:**
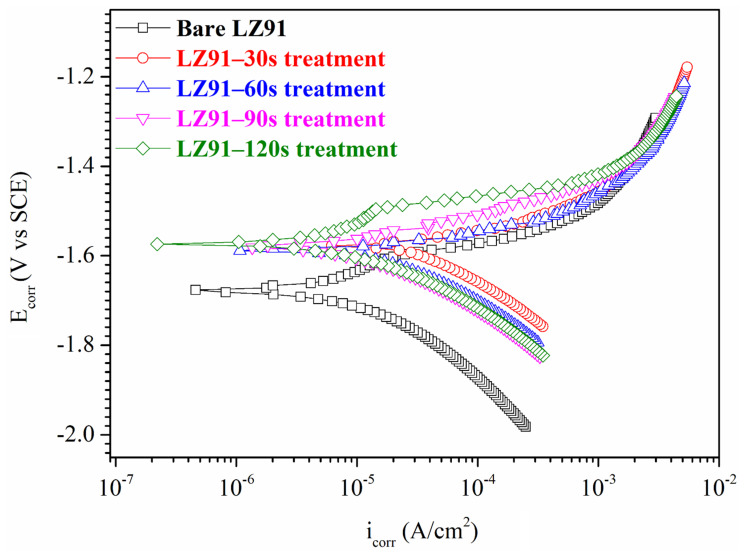
Potentiodynamic polarization curves for bare LZ91 and samples that undergo acid pickling pretreatment in 3.5 wt.% NaCl solution for different times.

The corrosion behavior of samples was determined by potentiodynamic techniques. Figure 5 and Table 1 show the polarization curves for bare LZ91 and for LZ91 plates that undergo acid pickling pretreatment. After the acid pickling treatment, the corrosion current density (*i_corr_*) for both the anodic and cathodic branches decreases, and the corrosion potential (*E_corr_*) is shifted in the noble direction. The cathodic current is caused by a decrease in dissolved oxygen or water, and the anodic current is caused by the oxidation and dissolution of the metal substrate or the coating. Figure 5 and Table 1 show that a sample that undergoes acid pickling pretreatment has a higher *E_corr_* (−1580 mV) than bare LZ91 (*E_corr_*: −1680 mV), but the corrosion current density is very similar.

### 3.2. The Properties of the Conversion Coating

**Figure 6:**
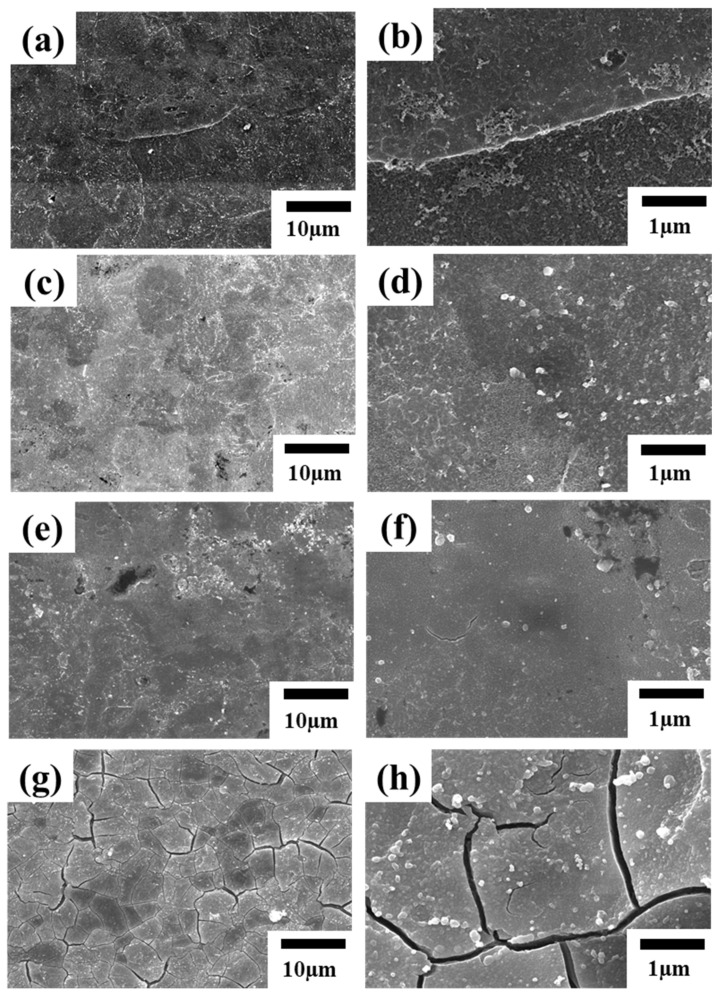
Surface morphology of LZ91 Mg alloy after conversion coating treatment for (**a**,**b**) 1 s, (**c**,**d**) 5 s, (**e**,**f**) 10 s, and (**g**,**h**) 30 s.

**Figure 7:**
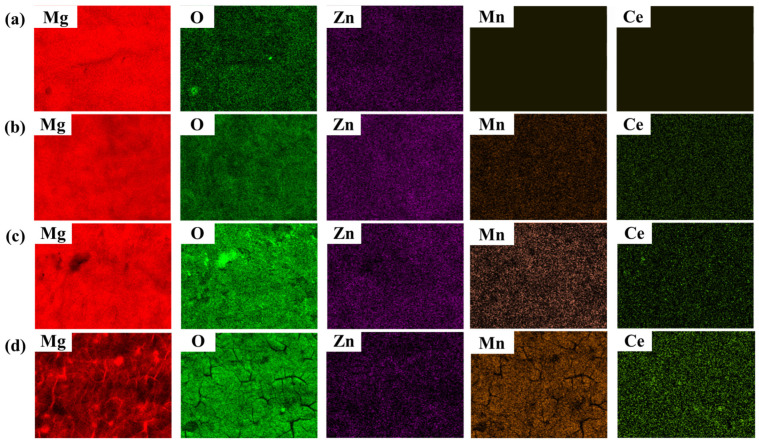
EDS/elemental mapping of the surface morphology image of LZ91 Mg alloy after conversion coating treatment for (**a**) 1 s, (**b**) 5 s, (**c**) 10 s, and (**d**) 30 s.

The results in Section 3.1 are for a conversion coating treatment using a fixed acid pickling pretreatment for 30 s. Figure 6 shows the surface morphology of the Mn-Ce conversion-coated LZ91 that is coated for 1 s, 5 s, 10 s, and 30 s. Figure 7 and Table 2 show the EDS analysis for the conversion coating. Figure 6 and Figure 7 show that for LZ91 immersed in the conversion coating for 1 s, no film is formed by the Mn-Ce on the surface: only the original substrate and magnesium oxide. Elemental Li cannot be detected by EDS. As the immersion time increases to 5 s, the specimen gradually forms a conversion coating, but the effect of the acid pickling pretreatment is still seen because the immersion time is short. As the immersion time increases to 10 s, the effect of acid pickling gradually disappears, and Figure 7 shows that the Mn and Ce contents gradually increase to form a conversion coating. As the immersion time increases to 30 s, a layer of conversion film is developed, and there are obvious cracks and some particles on the surface. The cracks form due to drying and dehydration [20,31,48,49].

**Figure 8:**
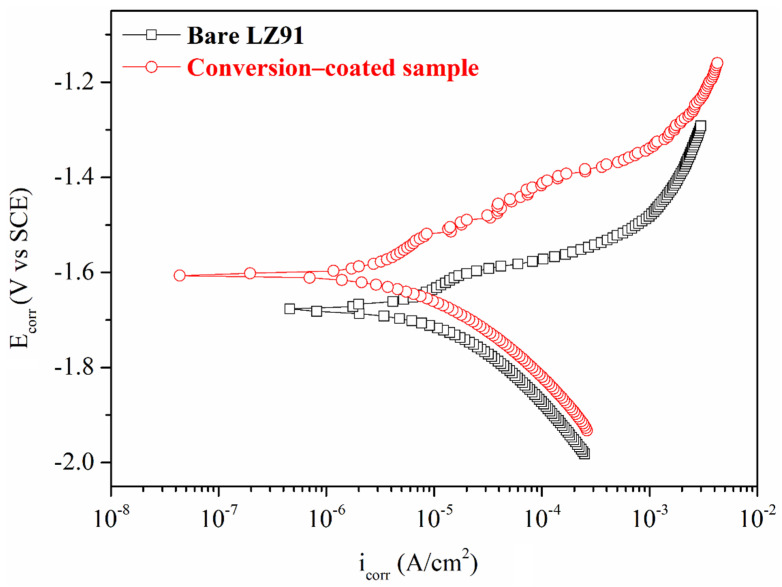
Potentiodynamic polarization curves for bare LZ91 and the conversion-coated sample using 3.5 wt.% NaCl solution.

Figure 8 and Table 3 show the polarization curves for bare LZ91 and for LZ91 plates with a conversion coating that uses 3.5 wt.% NaCl solution. The potentiodynamic polarization data are listed in Table 3. The average *i_corr_* values for bare LZ91 and LZ91 with a conversion coating are 10.2 μA·cm^−2^ and 4.57 μA·cm^−2^, respectively. The *E_corr_* value for the conversion coating is −1.68 V higher than that for the substrate (−1.58 V). When the conversion coating forms, the *i_corr_* value is decreased by almost one order of magnitude.

**Figure 9:**
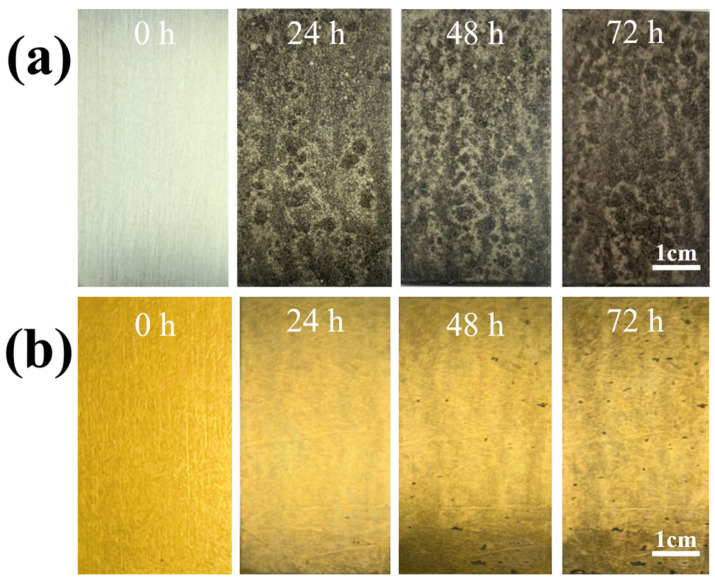
Optical images of (**a**) bare LZ91 and (**b**) the conversion-coated sample after 72 h SST.

Figure 9 shows the results of the SST after 72 h to verify the polarization curve. After 24 h of SST, the bare LZ91 is fully corroded. According to the ASTM D610-08 standard, LZ91 Mg alloy treated in the Mn-Ce solution for 30 s features minor points of oxidation on the surface after 72 h of SST.

### 3.3. The Formation Mechanism for the Conversion Coating

**Figure 10:**
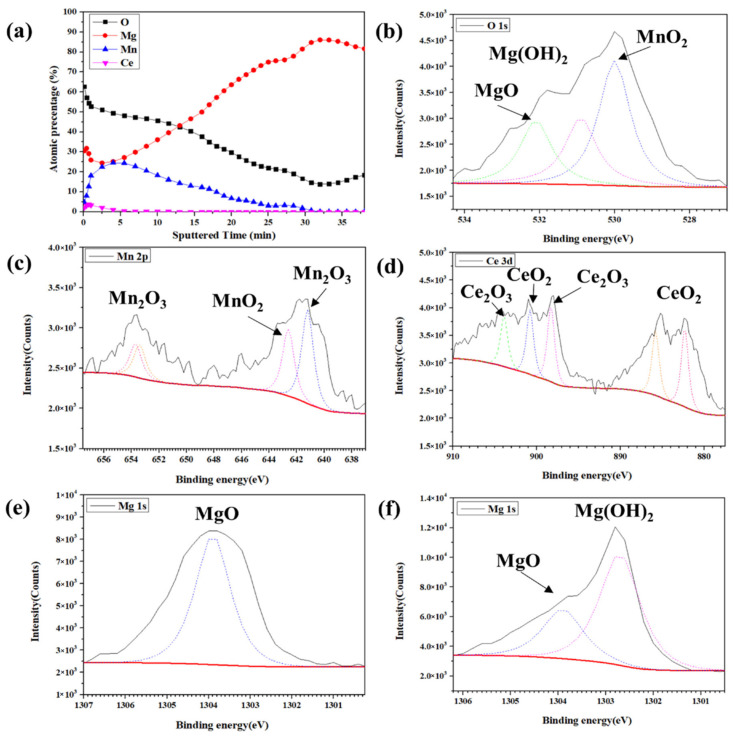
XPS analysis for the Mn-Ce conversion coating on LZ91 Mg alloy: (**a**) the depth profile and high-resolution XPS scan for (**b**) O 1s, (**c**) Mn 2p, (**d**) Ce 3d, (**e**) Mg 1s outer layer, and (**f**) Mg 1s inner layer.

The XPS spectrum for the conversion coating after immersion for 30 s was shown in Figure 10. In Figure 10a, the depth profile shows the element distribution in the Mn-Ce conversion coating during the etching of Ar^+^. The coating layer is composed of Mn, Ce, O, and Mg. This image shows that as the depth of the test piece increases, there is less manganese and oxygen, and the magnesium content increases. Ce only exists on the surface of the specimens. The XPS spectra for O 1s are shown in Figure 10b. There are three peaks at binding energies of 530.0, 530.9, and 532.1, corresponding to MnO_2_, Mg(OH)_2_, and MgO. Figure 10c shows that the high-resolution spectrum for Mn 2p can be deconvoluted into four peaks for Mn_2_O_3_ and MnO_2_, with a binding energy of 641.2, 642.6, 653.4, and 653.7 [19,50]. The Ce 3d peak is deconvoluted into five peaks at 882.3, 885.8, 898.3, 900.8, and 903.9 eV, as shown in Figure 10d, attributed to CeO_2_ and Ce_2_O_3_ [51]. The inner and outer conversion coatings contain two different types of Mg 1s at XPS. The outer Mg 1s peak is at 1303.9 eV, which is MgO, but the inner Mg 1s peak has two peaks at 1302.7 and 1303.9 eV, respectively, corresponding to Mg(OH)_2_ and MgO [19].

**Figure 11:**
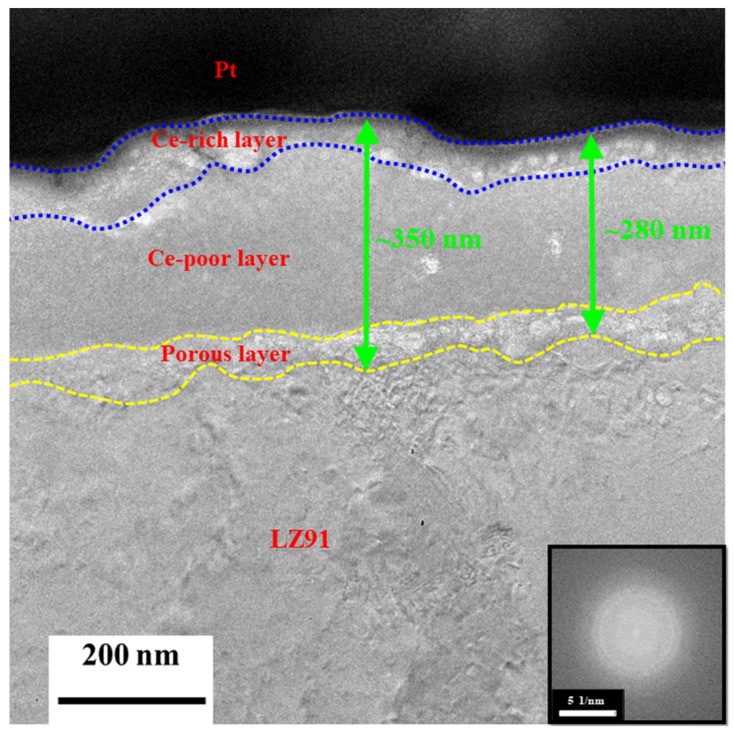
TEM image showing the cross-sectional morphology of the conversion coating on LZ91 Mg alloy.

**Figure 12:**
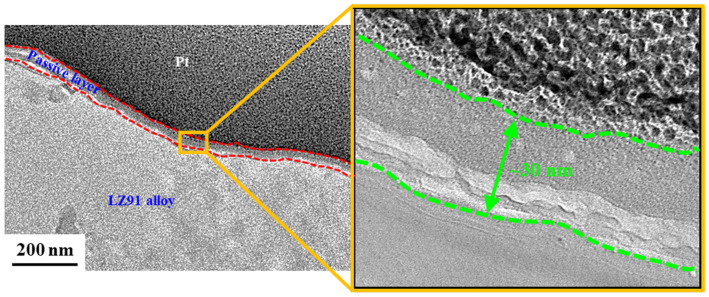
TEM image showing the cross-sectional morphology of LZ91 Mg alloy after acid pickling pretreatment for 30 s.

Figure 11 shows the cross-sectional TEM image for LZ91 Mg alloy after immersion in the Mn-Ce conversion coating for 30 s. This conversion coating has a double layer in the structure of LZ91 Mg alloy, which includes a relatively dense layer and a porous layer in contact with the LZ91 Mg substrate. The relatively dense layer also has Ce-rich and Ce-poor regions, consistent with the depth profile analysis for XPS (Figure 10a). The conversion coating is dispersed, and there are nano-voids. These nano-voids evolve when the LZ91 Mg substrate dissolves rapidly in the acidic solution and hydrogen is reduced, so MgO and Mg(OH)_2_ are formed. The average thickness of the conversion coating is approximately 315 nm. In our previous study [33], the thickness of the porous layer and overall layer (using grinding pretreatment) is between 100 nm and 650 nm, respectively. However, implementing the acid pickling pretreatment, the porous layer is thinner at only 60 nm. The overall thickness is reduced by half (from 650 nm to 315 nm). TEM image showing the cross-sectional morphology of LZ91 Mg alloy after acid pickling pretreatment for 30 s, as shown in Figure 12. It can be clearly found that a uniform passive layer is formed on the surface of LZ91 alloy. The average thickness of the passive layer is approximately 30 nm. Additionally, ICP-OES is used to compare the dissolution of LZ91 alloy after different pretreatment in the conversion coating solution. The dissolved amount of magnesium with grinding pretreatment and acid pickling pretreatment is 0.091 mg/L and 0.032 mg/L in the conversion coating solution, as shown in Table 4. In other words, the passive layer formed by acid pickling pretreatment can inhibit the dissolution of magnesium. This is the main reason for the thinning of the porous layer, which reduces the generation of the hydroxide/oxide layers (MgOH_2_/MgO).

**Figure 13:**
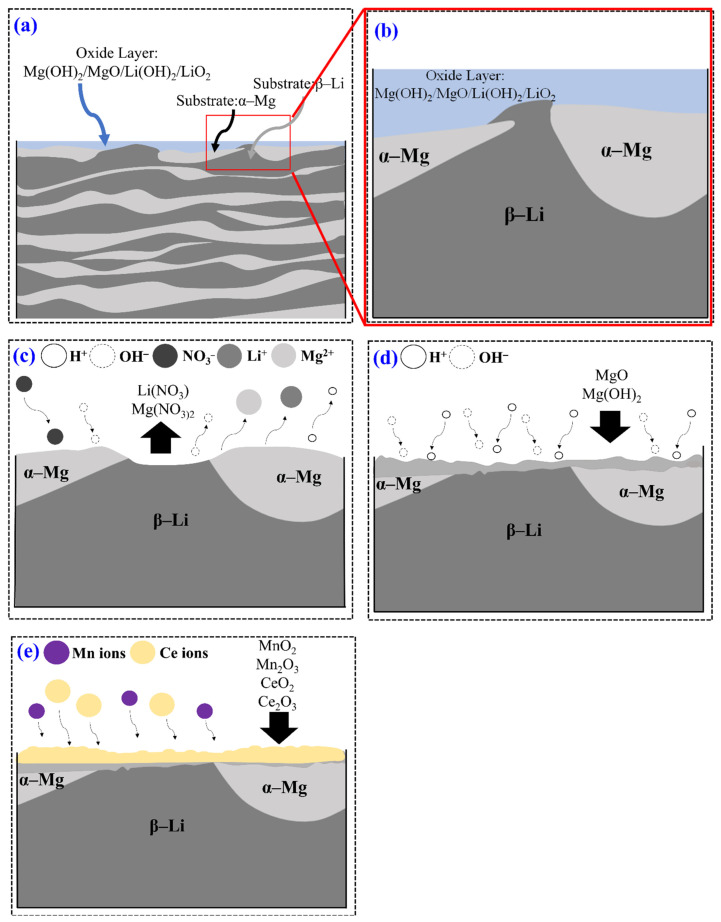
A schematic illustration of (**a**) initial LZ91 Mg alloy in natural surroundings, (**b**) partially enlarged image in (**a**), (**c**) acid pickling pretreatment, (**d**) initial immersion in conversion coating solution, and (**e**) the deposition of Mn-Ce conversion coating on LZ91 Mg alloy.

According to the above, the mechanism for the conversion coating treatment uses an acid pickling pretreatment, as shown in Figure 13. In natural surroundings, the LZ91 Mg alloy substrate spontaneously forms MgO/Mg (OH)_2_/Li_2_O/Li(OH), a very thin and unstable film, as shown in Figure 13a,b [52]. When LZ91 Mg alloy is immersed in the HNO_3_ solution, the Pourbaix diagram for magnesium shows that Mg dissolves into Mg^2+^ ions in the liquid phase, as shown in Equation (1). H^+^ ions in the solution then accept electrons and are reduced to H_2_(g), as shown in Equation (2). The magnesium and lithium hydroxides react with nitric acid to produce Mg(NO_3_)_2_ and LiNO_3_ as a precipitate, as shown in Equations (3) and (4) [52]. This process is shown in Figure 13c.

(1) $$\mathrm{Mg}\to {\mathrm{Mg}}^{2+}+2e^{-}$$

(2) $$2H^{+}+2e^{-}\;\to {\;H}_{2}$$

(3) $$\mathrm{Mg}{\left(\mathrm{OH}\right)}_{2}+{\;\mathrm{HNO}}_{3}\;\to \;\mathrm{Mg}{\left({\mathrm{NO}}_{3}\right)}_{2}+{\;H}_{2}O$$

(4) $$\mathrm{LiOH}\;+{\;\mathrm{HNO}}_{3}\;\to {\;\mathrm{LiNO}}_{3}+{\;H}_{2}O$$

When oxides and hydroxides on the surface react with nitric acid, the oxides and hydroxides on the surface of the test piece gradually dissolve, exposing a fresh substrate surface. When the substrate is exposed, magnesium reacts with nitric acid as [53]:

(5) $$\mathrm{Mg}\;+2{\mathrm{HNO}}_{3}\;\to \;\mathrm{Mg}{\left({\mathrm{NO}}_{3}\right)}_{2}+{\;H}_{2}$$

After acid pickling pretreatment, the LZ91 Mg alloy is exposed to the atmosphere and oxidized to MgO and Mg(OH)_2_ in order to protect the substrate from corrosion. During acid pickling pretreatment for LZ91 Mg alloy in the conversion coating solution, the oxide layer dissolves quickly as magnesium metal is dissolved. Stable Mg^2+^ is generated in the solution, and the excess of released electrons react with water and hydrogen ions, so there is a lack of hydrogen ions at the reaction interface and an increase in local pH. The concentration of hydrogen ions in the solution decreases, so the relative concentration of hydroxide ions increases. The anode reaction produces a large number of magnesium ions and lithium ions at the interface and magnesium hydroxide, which is barely soluble in water, so this is deposited on the surface, forming a looser porous layer (Equations (6)–(8)) [19].

(6) $${\mathrm{Mg}}^{+}+2H_{2}O\;+{\;e}^{-}\;\to {\;\mathrm{Mg}}^{2+}+{\;H}_{2}+2{\mathrm{OH}}^{-}$$

(7) $$2H^{+}+2e^{-}\;\to {\;H}_{2}$$

(8) $$2H_{2}O\;+2e^{-}\;\to {\;H}_{2}+2{\mathrm{OH}}^{-}$$

Heptavalent manganese ions are also directly reduced to a manganese dioxide precipitate, or heptavalent manganese ions are reduced to divalent manganese ions, and a Guyard reaction occurs, so Mn (II) ions and Mn (III) ions spontaneously react to produce manganese oxide (MnO_2_) and dimanganese trioxide (Mn_2_O_3_). The manganese dioxide is incorporated into the film structure (Equations (9)–(13)) [19,31,33,54].

(9) $${\mathrm{Mg}}^{2+}+2{\mathrm{OH}}^{-}\;\to \;\mathrm{Mg}{\left(\mathrm{OH}\right)}_{2}$$

(10) $$\mathrm{Mg}{\left(\mathrm{OH}\right)}_{2}\;\to {\;\mathrm{MgO}}_{\left(s\right)}+{\;H}_{2}O$$

(11) $${\mathrm{MnO}}_{4}^{-}+4H^{+}+3e^{-}\;\to {\;\mathrm{MnO}}_{2}+2H_{2}O$$

(12) $${\mathrm{MnO}}_{4}^{-}+8H^{+}+5e^{-}\;\to {\;\mathrm{Mn}}^{2+}+4H_{2}O$$

(13) $$2{\mathrm{MnO}}_{4}^{-}+3{\mathrm{Mn}}^{2+}+2H_{2}O\;\to \;5{\mathrm{MnO}}_{2}+4H^{+}$$

Cerium nitrate dissociates from Ce^3+^ in an aqueous solution and is almost oxidized to Ce^4+^ by potassium permanganate, which is a strong oxidant. In addition to reacting with magnesium ions, the hydroxide produced by the anode reaction forms Ce(III) and Ce(IV). Ce(OH)_3_ and Ce(OH)_4_ precipitate, and cerium(III) and (IV) hydroxide undergo a dehydration reaction due to subsequent drying (Equations (14)–(17)) [34,35].

(14) $${\mathrm{Ce}}^{3+}+3{\mathrm{OH}}^{-}\;\to \;\mathrm{Ce}{\left(\mathrm{OH}\right)}_{3}$$

(15) $${\mathrm{Ce}}^{4+}+4{\mathrm{OH}}^{-}\;\to \;\mathrm{Ce}{\left(\mathrm{OH}\right)}_{4}$$

(16) $$2\mathrm{Ce}{\left(\mathrm{OH}\right)}_{3}\;\to {\;\mathrm{Ce}}_{2}O_{3}+2H_{2}O$$

(17) $$\mathrm{Ce}{\left(\mathrm{OH}\right)}_{4}\;\to {\;\mathrm{CeO}}_{2}+2H_{2}O$$

## 4. Conclusions

This study conducts an acid pickling pretreatment for different times in nitric acid solution to determine the effect on roughness, contact angle, corrosion resistance, and microstructure. A Mn-Ce conversion coating on LZ91 Mg alloy increases corrosion resistance. Acid pickling pretreatment removes oxides, and a Mn-Ce conversion coating on LZ91 Mg alloy immersed for 30 s is thin and compact. The Mn-Ce conversion coating on LZ91 Mg alloy that is formed after immersion for 30 s is composed of Mg, O, Mn, and Ce, as shown by EDS in SEM. The Mn content of the conversion coating is at 12.7%, and the Ce content is at 0.9%. The main compounds that are detected by XPS are MnO_2_, Mn_2_O_3_, CeO_2_, and Ce_2_O_3_. The conversion coating has a thickness of 450 nm, as shown by TEM. The porous layer produced by pickling is thinner (60 nm) than that produced by grinding (100 nm). The corrosion resistance of a Mn-Ce conversion coating reduces the surface area of corrosion to less than 5% after 72 h SST, and the *i_corr_* value can decrease by nearly one order (from 10.2 to 4.57 μA·cm^−2^), which means 2.25 times better corrosion resistance.

## Figures and Tables

**Table 1:** Results for potentiodynamic polarization tests for bare LZ91 and the samples that undergo acid pickling pretreatment in 3.5 wt.% NaCl solution for different times.

Specimen	*i_corr_* (μA·cm^−2^)	*E_corr_* (V)
Bare LZ91	10.2 (±1.35)	−1.68 (±5.21 × 10^−3^)
30 s	44.0 (±2.71)	−1.58 (±6.46 × 10^−3^)
60 s	22.0 (±2.34)	−1.59 (±4.51 × 10^−3^)
90 s	14.5 (±2.01)	−1.58 (±5.19 × 10^−3^)
120 s	11.9 (±1.81)	−1.57 (±5.34 × 10^−3^)

**Table 2:** Results for EDS for LZ91 Mg alloy after conversion coating treatment.

Specimen	Mg	O	Zn	Mn	Ce	Others
1 s	94	5.6	0.4	0	0	0
5 s	87.5	10.5	0.3	1.3	0.3	0.1
10 s	80.6	16.3	0.3	2.5	0.4	0
30 s	45.8	39.7	0.3	12.7	0.9	0.6

**Table 3:** Results for potentiodynamic polarization tests for bare LZ91 and the conversion-coated sample.

Specimen	*i_corr_* (μA·cm^−2^)	*E_corr_* (V)
Bare LZ91	10.2 (±1.35)	−1.68 (±5.21 × 10^−3^)
Conversion-coated sample	4.57 (±1.45)	−1.58 (±5.15 × 10^−3^)

**Table 4:** Dissolved amount of magnesium with different pretreatment in the conversion coating solution.

Specimen	Dissolved Amount of Magnesium (mg/L)
Grinding pretreatment	0.091 (±0.004)
Acid pickling pretreatment	0.032 (±0.002)

## Data Availability

No new data were created or analyzed in this study.

## References

[1] Haferkamp H., Boehm R., Holzkamp U., Jaschik C., Kaese V., Niemeyer M. (2001). Alloy Development, Processing and Applications in Magnesium Lithium Alloys. Mater. Trans..

[2] Sanschagrin A., Tremble R., Angers R., Dubé D. (2001). Mechanical properties and microstructure of new magnesium–Lithium base alloys. Mater. Sci. Eng. A.

[3] Wu R., Qu Z., Zhang M. (2009). Effects of the addition of Y in Mg–8Li– (1,3) Al alloy. Mater. Sci. Eng. A.

[4] Xu T., Yang Y., Peng X., Song J., Pan F. (2019). Overview of advancement and development trend on magnesium alloy. J. Magnes. Alloy..

[5] Braga D.F.O., Tavares S.M.O., da Silva L.F.M., Moreira P.M.G.P., de Castro P.M.S.T. (2014). Advanced design for lightweight structures: Review and prospects. Prog. Aerosp. Sci..

[6] Yang H., Guo X., Chen X., Birbilis N. (2014). A homogenisation pre-treatment for adherent and corrosion resistant Ni electroplated coatings on Mg-alloy AZ91D. Corros. Sci..

[7] Singh C., Tiwari S.K., Singh R. (2021). Development of corrosion-resistant electroplating on AZ91 Mg alloy by employing air and water-stable eutectic based ionic liquid bath. Surf. Coat. Technol..

[8] Yin T.T., Wu R., Leng Z., Du G., Guo X., Zhang M., Zhang J.H. (2013). The process of electroplating with Cu on the surface of Mg-Li alloy. Surf. Coat. Technol..

[9] Ghavidel N., Allahkaram S.R., Nederi R., Barzegar M., Bakhshandeh H. (2020). Corrosion and wear behavior of an electroless Ni-P/nano-SiC coating on AZ31 Mg alloy obtained through environmentally-friendly conversion coating. Surf. Coat. Technol..

[10] Zhang H., Wang S., Yao G., Hua Z. (2009). Electroless Ni–P plating on Mg–10Li–1Zn alloy. J. Alloys Compd..

[11] Yang L., Li J., Zheng Y., Jiang W., Zheng M. (2009). Electroless Ni–P plating with molybdate pretreatment on Mg–8Li alloy. J. Alloys Compd..

[12] Jin J., Liu C., Fu S., Guo Y., Shu X. (2011). Electroless Ni-P plating on Mg-10Gd-4.8Y-0.6Zr magnesium alloy with a new pretreatment process. Surf. Coat. Technol..

[13] Li J.F., Zheng Z.Q., Li S.C., Ren W.D., Zheng Z. (2006). Preparation and galvanic anodizing of a Mg-Li alloy. Mater. Sci. Eng. A.

[14] Garcia-Vergara S.J., Skeldon P., Thompson G.E., Habazaki H. (2006). A flow model of porous anodic film growth on aluminum. Electrochim. Acta.

[15] Wu G., Zeng X., Ding W., Guo X., Yao S. (2006). Characterization of ceramic PVD thin films on AZ31 magnesium alloys. Appl. Surf. Sci..

[16] Chen X.B., Chong K., Abbott T.B., Birbilis N., Easton M.A. (2015). 15-Biocompatible strontium-phosphate and manganese-phosphate conversion coatings for magnesium and its alloys. Surface Modification of Magnesium and Its Alloys for Biomedical Applications.

[17] Doerre M., Hibbitts L., Patrick G., Akafuah N.K. (2018). Advances in Automotive Conversion Coatings during Pretreatment of the Body Structure: A Review. Coatings.

[18] Jian S.Y., Chu Y.R., Lin C.S. (2015). Permanganate conversion coating on AZ31 magnesium alloys with enhanced corrosion resistance. Corros. Sci..

[19] Hung S.M., Lin H., Chen H.W., Chen S.Y., Lin C.S. (2021). Corrosion resistance and electrical contact resistance of a thin permanganate conversion coating on dual-phase LZ91 Mg–Li alloy. J. Mater. Res. Technol..

[20] Zucchi F., Frignani A., Grassi V., Trabanelli G., Monticelli C. (2007). Stannate and permanganate conversion coatings on AZ31 magnesium alloy. Corros. Sci..

[21] Zhang H., Yao G., Wang S., Liu Y., Luo H. (2008). A chrome-free conversion coating for magnesium–lithium alloy by a phosphate–permanganate solution. Surf. Coat. Technol..

[22] Lee Y.L., Chu Y.R., Li W.C., Lin C.S. (2013). Effect of permanganate concentration on the formation and properties of phosphate/permanganate conversion coating on AZ31 magnesium alloy. Corros. Sci..

[23] Song Y., Shan D., Chen R., Zhang F., Han E.H. (2009). Formation Mechanism of Phosphate Conversion Film on Mg-8.8 Li Alloy. Corros. Sci..

[24] Wu Q., Yu B., Zhou P., Zhang T., Wang F. (2021). Fabrication of phosphate conversion coatings on rolled AZ31 magnesium alloy: Variation of corrosion resistance on different planes induced by the crystallographic texture. Mater. Chem. Phys..

[25] Liao S., Yu B., Zhang B., Zhou P., Zhang T., Wang F. (2021). Chemically depleting the noble impurities from AZ91-T4 magnesium alloy: A new and efficient pretreatment method to improve the corrosion resistance of phosphate conversion coatings. Corros. Sci..

[26] Lin C.S., Lee C.Y., Li W.C., Chen Y.S., Fang G.N. (2006). Formation of Phosphate/Permanganate Conversion Coating on AZ31 Magnesium Alloy. J. Electrochem. Soc..

[27] Wang G., Zhang M., Wu R. (2012). Molybdate and molybdate/permanganate conversion coatings on Mg–8.5 Li alloy. Appl. Surf. Sci..

[28] Yang L., Zhang M., Li J., Yu X., Niu Z. (2009). Stannate conversion coatings on Mg–8Li alloy. J. Alloys Compd..

[29] Lee Y.L., Chu Y.R., Chen F.J., Lin C.S. (2013). Mechanism of the formation of stannate and cerium conversion coatings on AZ91D magnesium alloys. Appl. Surf. Sci..

[30] Guo L., Zhang F., Song L., Zeng R.C., Li S.Q., Han E.H. (2017). Corrosion resistance of ceria/polymethyltrimethoxy silane modified magnesium hydroxide coating on AZ31 magnesium alloy. Surf. Coat. Technol..

[31] Montemor M., Simoes A., Carmezim M. (2007). Characterization of rare-earth conversion films formed on the AZ31 magnesium alloy and its relation with corrosion protection. Appl. Surf. Sci..

[32] Rudd A.L., Breslin C.B., Mansfeld F. (2000). The corrosion protection afforded by rare earth conversion coatings applied to magnesium. Corros. Sci..

[33] Jian S.Y., Chang K.L. (2020). Effect of cerium ion on the microstructure and properties of permanganate conversion coating on LZ91 magnesium alloy. Appl. Surf. Sci..

[34] Jian S.Y., Tzeng Y.C., Ger M.D., Chang K.L., Shi G.N., Huang W.H., Chen C.Y., Wu C.C. (2020). The study of corrosion behavior of manganese-based conversion coating on LZ91 magnesium alloy: Effect of addition of pyrophosphate and cerium. Mater. Des..

[35] Jian S.Y., Yang C.Y., Chang J.K. (2020). Robust corrosion resistance and self-healing characteristics of a novel Ce/Mn conversion coatings on EV31 magnesium alloys. Appl. Surf. Sci..

[36] Höche D., Nowak A., John-Schillings T. (2013). Surface cleaning and pre-conditioning surface treatments to improve the corrosion resistance of magnesium (Mg) alloys. Corrosion Prevention of Magnesium Alloy.

[37] Yuan J., Li P., Yuan R., Mao D., Zhao S., Feng T. (2020). Influence of pickling time on electroless Ni–P coating on magnesium alloy. Mater. Corros.-Werkst. Korros..

[38] Elsentriecy H.H., Azumi K., Konno H. (2007). Effect of surface pretreatment by acid pickling on the density of stannate conversion coatings formed on AZ91 D magnesium alloy. Surf. Coat. Technol..

[39] Su H.Y., Li W.J., Lin C.S. (2012). Effect of Acid Pickling Pretreatment on the Properties of Cerium Conversion Coating on AZ31 Magnesium Alloy. J. Electrochem. Soc..

[40] Dong X. (2015). Surface Treatments for Magnesium Alloys.

[41] Song D., Jing X., Wang J., Lu S., Yang P., Wang Y., Zhang M. (2011). Microwave-assisted synthesis of lanthanum conversion coating on Mg–Li alloy and its corrosion resistance. Corros. Sci..

[42] Yang H.Y., Chen X.B., Guo X.W., Wu G.H., Ding W.J., Birbilis N. (2012). Coating pretreatment for Mg alloy AZ91D. Appl. Surf. Sci..

[43] Jiang X., Guo R., Jiang S. (2016). Evaluation of self-healing ability of Ce–V conversion coating on AZ31 magnesium alloy. J. Magnes. Alloys.

[44] Yang X., Wang G., Dong G., Gong F., Zhang M. (2009). Rare earth conversion coating on Mg–8.5 Li alloys. J. Alloys Compd..

[45] (2022). Practice for Operating Salt Spray (Fog) Apparatus, ASTM Volume 03.02: Corrosion of Metals; Wear and Erosion.

[46] (2022). Practice for Evaluating Degree of Rusting on Painted Steel Surfaces, ASTM Volume 06.01: Paint—Tests for Chemical, Physical, and Optical Properties; Appearance.

[47] Lee Y.L., Chen F.J., Lin C.S. (2013). Corrosion Resistance Studies of Cerium Conversion Coating with a Fluoride-Free Pretreatment on AZ91D Magnesium Alloy. J. Electrochem. Soc..

[48] Chong K.Z., Shih T.S. (2003). Conversion-coating treatment for magnesium alloys by a permanganate–phosphate solution. Mater. Chem. Phys..

[49] Zhou W., Shan D., Han E.H., Ke W. (2008). Structure and formation mechanism of phosphate conversion coating on die-cast AZ91D magnesium alloy. Corros. Sci..

[50] Chen X.B., Zhou X., Abbott T.B., Easton M.A., Birbilis N. (2013). Double-layered manganese phosphate conversion coating on magnesium alloy AZ91D: Insights into coating formation, growth and corrosion resistance. Surf. Coat. Technol..

[51] Wang H., Li Y., Wang F. (2008). Influence of cerium on passivity behavior of wrought AZ91 alloy. Electrochim. Acta.

[52] Sun Y.H., Wang R.C., Peng C.Q., Feng Y., Yang M. (2017). Corrosion behavior and surface treatment of superlight Mg−Li alloys. Trans. Nonferrous Met. Soc. China.

[53] Ohara M., Okahara H., Takigawa Y., Higashi K. (2008). Clarification of the Necessary Value of Surface Roughness for Developing Luster on an AZ31 Magnesium Alloy Surface with or without Acid Aqueous Solution Treatment. Mater. Trans..

[54] Polissar M.J. (1935). The Kinetics of the Reaction between Permanganate and Manganous Ions. J. Phys. Chem..

